# Predictive role of ABC transporters in the efficacy of enfortumab vedotin for urothelial carcinoma

**DOI:** 10.1002/bco2.488

**Published:** 2025-01-11

**Authors:** Toshiki Kijima, Atsuko Takada‐Owada, Hiroki Shimoda, Hidetoshi Kokubun, Toshitaka Uematsu, Kohei Takei, Hironori Betsunoh, Masahiro Yashi, Kazuyuki Ishida, Takao Kamai

**Affiliations:** ^1^ Department of Urology Dokkyo Medical University Tochigi Japan; ^2^ Department of Diagnostic Pathology Dokkyo Medical University Tochigi Japan; ^3^ Department of Urology Tokyo Medical and Dental University, Dokkyo Medical University Tokyo Japan

**Keywords:** ABC transporters, drug resistance, Enfortumab Vedotin, metastatic urothelial carcinoma, Nectin‐4

## Abstract

**Objective:**

To evaluate the correlation between ATP‐binding cassette (ABC) transporter expression and therapeutic efficacy of enfortumab vedotin (EV), an antibody‐drug conjugate targeting Nectin‐4, in urothelial cancer, as only a few studies have been conducted on this topic.

**Patients and methods:**

This retrospective study included 20 patients with metastatic urothelial carcinoma (mUC), including bladder and upper urinary tract cancers, who were treated with EV at Dokkyo Medical University Hospital between 2022 and 2024. Immunohistochemical staining was performed on formalin‐fixed, paraffin‐embedded tissue samples. Progression‐free survival (PFS) was estimated using the Kaplan–Meier method, and differences between subgroups (e.g., Nectin‐4 and ABC transporter expression) were compared using the log‐rank test.

**Results:**

Immunohistochemical analysis indicated that tumours with high ABC transporter expression exhibited shorter PFS time and poorer response to EV. Furthermore, a decrease in Nectin‐4 expression and an increase in ABC transporter expression were observed as the disease progressed from non‐muscle‐invasive to muscle‐invasive and metastatic. Patients with Nectin‐4‐positive and ABC transporter‐negative tumours had the longest PFS, underscoring the prognostic significance of these biomarkers.

**Conclusion:**

To our knowledge, this study is the first to show a correlation between ABC transporter expression and EV efficacy in urothelial carcinoma. Future research should focus on optimizing treatment strategies based on Nectin‐4 and ABC transporter expression to improve outcomes.

## 1 INTRODUCTION

Bladder cancer is one of the most prevalent malignancies of the urinary system, and metastatic urothelial carcinoma (mUC) remains a disease with a poor prognosis. The standard treatment for bladder cancer is platinum‐based chemotherapy, particularly cisplatin, and immune checkpoint inhibitors, such as pembrolizumab. However, many patients exhibit resistance or intolerance to these therapies, which limits the available therapeutic options. In recent years, antibody‐drug conjugates (ADCs) have emerged as a promising new treatment for metastatic bladder cancer.

Enfortumab vedotin (EV) is a groundbreaking ADC therapy targeting metastatic bladder cancer, specifically designed to bind to Nectin‐4, a cell adhesion molecule highly expressed in urothelial carcinoma. EV links a monoclonal antibody targeting Nectin‐4 with the cytotoxic agent monomethyl auristatin E (MMAE), selectively delivering the drug to cancer cells and inducing cell death. Clinical trials have revealed that EV has a higher response rate than traditional chemotherapy, particularly in patients with mUC who have progressed after treatment with immune checkpoint inhibitors.^1^ However, the efficacy of EV is not uniform across all patients, and the biological characteristics of individual tumours influence its effectiveness. One of the key factors affecting the efficacy of EV is the expression level of Nectin‐4, with previous studies showing that tumours with low Nectin‐4 expression exhibit reduced responses to EV.^2^ Nevertheless, the impact of factors other than Nectin‐4 on EV sensitivity remains largely unexplored.

Recently, attention has been paid to the role of ATP‐binding cassette (ABC) transporters in drug resistance mechanisms in cancer therapy. ABC transporters are proteins that utilize ATP to transport various substances across cell membranes and are known to contribute to drug resistance by exporting anticancer drugs from cells.^3^ Notably, MDR1 (ABCB1, P‐glycoprotein), MRP1 (ABCC1) and BCRP (ABCG2) are major transporters that mediate multidrug resistance in cancer cells by reducing intracellular drug concentrations, thereby diminishing the therapeutic effects of chemotherapeutic agents.^4^ In bladder cancer, overexpression of ABC transporters has been associated with resistance to chemotherapeutic drugs such as cisplatin and doxorubicin.^5^ However, few studies have been conducted on the relationship between ABC transporter expression and EV efficacy.

The aim of this study was to evaluate the correlation between ABC transporter expression and the therapeutic efficacy of EV in urothelial cancer. This will provide novel insights into prognostic factors and potential strategies for treatment optimization in mUC.

## 2 PATIENTS AND METHODS

### 2.1 Ethics statements

This retrospective study was approved by the institutional review board of Dokkyo Medical University (approval number: R‐31‐10 J) and adhered to the ethical guidelines of the Declaration of Helsinki. The protocol was publicly disclosed, and informed consent was obtained from all patients before inclusion in the study.

### 2.2 Patients

Data of 20 patients diagnosed with mUC who received EV therapy at Dokkyo Medical University Hospital between 2022 and 2024 were retrospectively analysed. All patients had previously received platinum‐based chemotherapy and immune checkpoint inhibitors (ICIs), and disease progression was confirmed before initiating EV therapy. Archived pathological specimens were obtained using the following surgical procedures: transurethral resection of bladder tumours (TURBT) for non‐muscle‐invasive tumours (n = 14), nephroureterectomy or radical cystectomy for muscle‐invasive tumours (n = 11) or metastasectomy for metastatic lesions (n = 8). Specimens were selected based on the availability of well‐preserved formalin‐fixed, paraffin‐embedded (FFPE) tissue samples for subsequent immunohistochemical (IHC) analysis.

### 2.3 Immunohistochemical staining

IHC staining was performed on FFPE tissue sections using the following primary antibodies: Nectin‐4 (EPR15613–68, Abcam, dilution 1:100), MDR1 (P‐glycoprotein, ABCB1) (EPR10364–57, Abcam, dilution 1:100), MRP1 (ABCC1) (EPR21062, Abcam, dilution 1:100) and BCRP (ABCG2) (BXP‐21, Abcam, dilution 1:50). IHC staining was conducted using an autoimmunostainer (Leica BOND‐III system: Leica Biosystems, Newcastle, UK). IHC staining was scored according to a modified version of the HER2 IHC scoring system.^6^ The scoring criteria were as follows: IHC score 0: no staining or incomplete and faint/barely perceptible membrane staining in ≤10% of the tumour cells. IHC score 1+: incomplete and faint/barely perceptible membrane staining in >10% of tumour cells. IHC score 2+: weak/moderate complete membrane staining in >10% of tumour cells or complete and intense membrane staining in ≤10% of tumour cells. IHC score 3+: complete and intense membrane staining in >10% of tumour cells. For Nectin‐4 and ABC transporter expression, samples with an IHC score ≥2 were considered positive, whereas those with a score ≤1 were considered negative. Regarding ABC transporters (ABCB1, MRP1 and BCRP), samples were classified as ABC transporter‐positive if any of the three transporters were positive. Samples in which all three transporters were negative were classified as ABC transporter‐negative.

To prevent bias, all slides were evaluated by a single pathologist (ATO) blinded to the patients' clinical information. Analysis of progression‐free survival (PFS) and other clinical outcomes was conducted by the principal investigator (TK), who was aware of the patient's clinical details.

### 2.4 Data collection and analysis

PFS was defined as the time from initiation of EV therapy to disease progression, death due to urothelial carcinoma, or last follow‐up. PFS was estimated using the Kaplan–Meier method, and differences between subgroups (e.g., Nectin‐4 and ABC transporter expression) were compared using the log‐rank test. All statistical analyses were conducted using the JMP software (version 13.0; SAS Institute). Statistical significance was set at P < 0.05. Owing to the small sample size and number of events, we did not perform multivariable Cox proportional hazards analysis in this study.

## 3 RESULTS

### 3.1 Patient characteristics

Patient characteristics at the initiation of EV therapy are summarized in Table 1. The median patient age was 70 (range, 45–84) years. The primary tumour sites were the upper urinary tract in eight patients (40%) and the bladder in 12 patients (60%). Synchronous metastases were observed in three patients (15%), while 17 patients (85%) had metachronous metastases. At the time of EV therapy initiation, metastatic sites included lymph nodes in 13 patients (65%), lungs in 10 patients (50%), the liver in five patients (25%), bone in three patients (15%) and adrenal glands in one patient (5%). Prior treatments included avelumab maintenance therapy in six patients and pembrolizumab in 14 patients. Tissue samples from non‐muscle‐invasive primary tumours were available for 14 patients, while muscle‐invasive tumours were available for 11 patients. Metastatic lesions were sampled in eight patients, with resected tissues obtained from lymphadenectomy (n = 1), bronchoscopy (n = 1), lung resection (n = 6) and adrenalectomy (n = 1).

**TABLE 1:** Patient characteristics.

	Variables	Median (range) or N (%)
Age at EV therapy		70 (45–84)
Primary tumour location	Upper tract	8 (40%)
	Bladder	12 (60%)
Development of metastases	Synchronous	3 (15%)
	Metachronous	17 (85%)
Metastatic sites at EV therapy	Distal lymph nodes	13 (65%)
	Lung	10 (50%)
	Liver	5 (25%)
	Bone	3 (15%)
	Adrenal	1 (5%)
Systemic therapy before EV therapy	GC/GCarbo	20 (100%)
	Avelumab maintenance	6 (30%)
	Pembrolizumab	14 (70%)
Method for obtaining non‐invasive tumour tissue	TUR‐Bt	12 (60%)
	Trans‐ureteral biopsy	2 (10%)
	Not performed	6 (30%)
Method for obtaining invasive tumour tissue	Nephroureterectomy	5 (25%)
	Radical/partial cystectomy	6 (30%)
	Not performed	9 (45%)
Method for obtaining metastatic tumour tissue	Lymph adenectomy	1 (5%)
	Bronchoscopic biopsy	1 (5%)
	Partial pneumonectomy	6 (30%)
	Adrenalectomy	1 (5%)
	Not performed	12 (60%)

### 3.2 Comparison of Nectin‐4 and ABC transporter expression between non‐muscle‐invasive and muscle‐invasive Tumours

**FIGURE 1:**
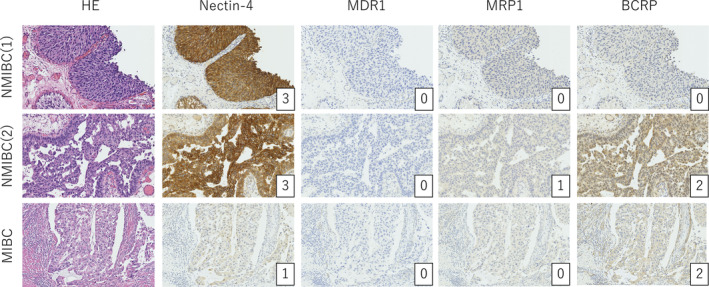
Comparison of Nectin‐4 and ABC transporter expression in non‐muscle‐invasive and muscle‐invasive bladder cancer. Immunohistochemical analysis of Nectin‐4 and ABC transporter (MDR1, MRP1, BCRP) expression in primary tumour samples from a representative case, comparing non‐muscle‐invasive bladder cancer (NMIBC) and muscle‐invasive bladder cancer (MIBC). Magnification: ×200.

Comparative analysis of Nectin‐4 and ABC transporter expression between non‐muscle‐invasive and muscle‐invasive primary tumours was possible in five cases (Table 2, Figure 1). In all five cases, Nectin‐4 expression decreased as the tumour progressed from non‐muscle‐invasive to muscle‐invasive disease. While no significant changes in ABC transporter expression were observed in most cases, MRP1 and BCRP expressions increased in one case. Figure 1 presents the immunohistochemical results from Case 4 in Table 2, where Nectin‐4 expression decreased from 3 + to 1 + .

**TABLE 2:** Comparison of Nectin‐4 and ABC transporter expression in non‐invasive and invasive primary tumours.

Case	Nectin‐4		MDR1		MRP1		BCRP	
	Non‐invasive	Invasive	Non‐invasive	Invasive	Non‐invasive	Invasive	Non‐invasive	Invasive
1	3	2	0	0	0	1	1	1
2	3	2	0	0	0	0	0	0
3	3	1	0	0	1	1	1	1
4	3	1	0	0	1	0	2	2
5	3	1	0	0	2	3	0	1

### 3.3 Comparison of Nectin‐4 and ABC transporter expression between primary and metastatic lesions

**FIGURE 2:**
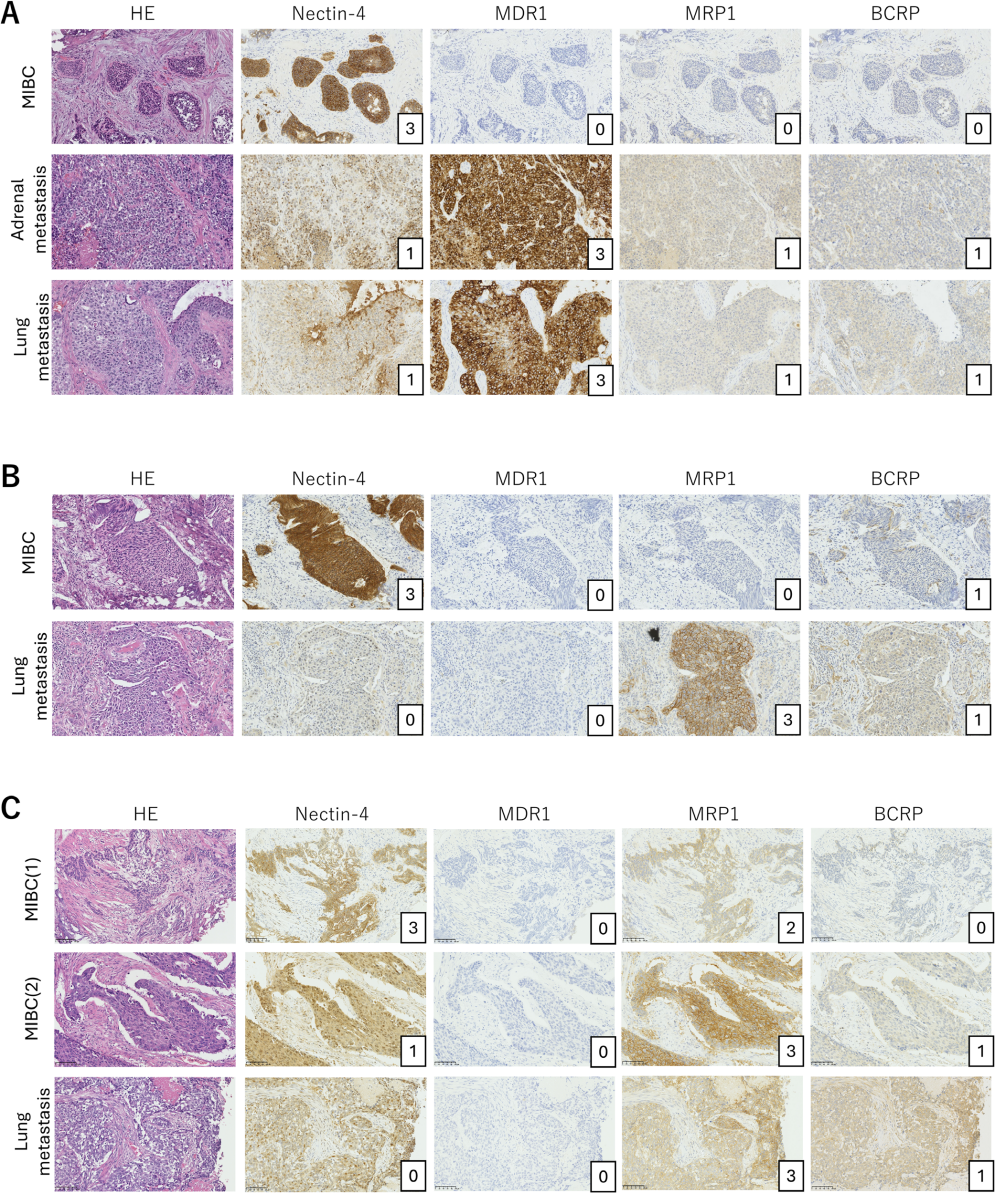
Comparison of Nectin‐4 and ABC transporter expression between primary tumours and metastatic lesions. Immunohistochemical analysis comparing Nectin‐4 and ABC transporter (MDR1, MRP1 and BCRP) expression between primary bladder tumours and metastatic lesions in three representative cases. Magnification: ×200.

In eight cases, we compared Nectin‐4 and ABC transporter expression between primary and metastatic lesions (Table 3, Figure 2). Nectin‐4 expression decreased in five out of eight metastatic lesions compared with that in primary tumours. MDR1 expression increased in two of eight cases, MRP1 expression increased in three of eight cases and BCRP expression increased in two of eight cases. Figure 2A shows the immunohistochemical results of case 2 from Table 3, where Nectin‐4 expression decreased from 3 + to 1 + and MDR1 expression increased from 0 to 3+. Figure 2B shows case 4, in which Nectin‐4 expression decreased from 3 + to 0 and MRP1 expression increased from 0 to 3+. Figure 2C shows case 6, in which Nectin‐4 expression decreased from 3 + to 0, MRP1 expression increased from 2 + to 3+ and BCRP expression increased from 0 to 1 + .

**TABLE 3:** Comparison of Nectin‐4 and ABC transporter expression in primary and metastatic tumours.

Case	Nectin‐4		MDR1		MRP1		BCRP	
	Primary	Metastasis	Primary	Metastasis	Primary	Metastasis	Primary	Metastasis
1	1	3	0	0	1	1	2	2
2	3	1	0	3	1	1	1	2
3	3	3	0	0	1	1	1	1
4	3	0	0	1	0	3	1	1
5	0	0	0	0	1	1	1	1
6	3	2	0	0	0	0	1	1
7	3	0	0	0	2	3	0	1
8	3	2	0	0	0	1	1	1

### 3.4 Expression of Nectin‐4 and ABC transporters in non‐muscle‐invasive tumour, muscle‐invasive tumour and metastatic lesions

Figure S1 shows the expression frequency of Nectin‐4 and ABC transporters (MDR1, MRP1 and BCRP) in non‐muscle‐invasive tumours (n = 14), muscle‐invasive tumours (n = 11) and metastatic lesions (n = 8). The figure illustrates that Nectin‐4 expression decreases as the tumour progresses from non‐muscle‐invasive to muscle‐invasive disease, and further decreases in metastatic lesions. In contrast, ABC transporter expression increased as the tumour progressed, indicating a possible correlation between tumour progression and resistance mechanisms.

### 3.5 Impact of Nectin‐4 and ABC transporter expression on PFS after EV therapy

**FIGURE 3:**
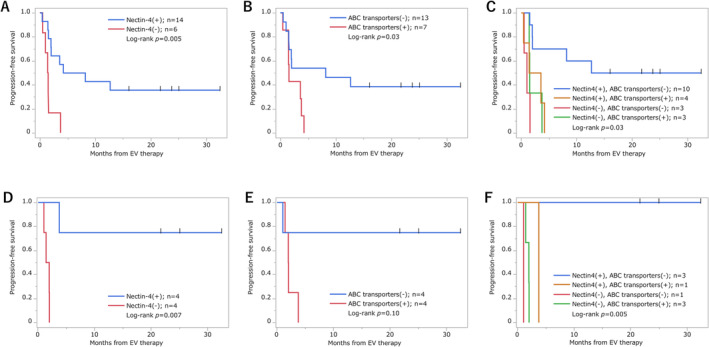
Impact of Nectin‐4 and ABC transporter expression on progression‐free survival after Enfortumab Vedotin therapy. A: PFS according to Nectin‐4 expression in primary tumours. B: PFS by ABC transporter expression in primary tumours. C: PFS by combined Nectin‐4 and ABC transporter expression in primary tumours. D: PFS by Nectin‐4 expression in metastatic lesions. E: PFS by ABC transporter expression in metastatic lesions. F: PFS by combined Nectin‐4 and ABC transporter expression in metastatic lesions.

We evaluated the association between Nectin‐4 and ABC transporter expression in primary tumour samples, including both non‐muscle‐invasive and muscle‐invasive tumours, and PFS after EV therapy in all 20 patients. For cases in which both non‐muscle‐invasive and muscle‐invasive tumour samples were available, the results in the muscle‐invasive sample were used in the following analyses. Patients with Nectin‐4‐positive tumours had significantly longer PFS, compared with those with Nectin‐4‐negative tumours (median PFS: 6.2 months vs. 1.4 months, respectively; p = 0.005, Figure 3A). Similarly, patients with ABC transporter‐negative tumours had significantly longer PFS, compared with those with ABC transporter‐positive tumours (median PFS: 8.2 months vs. 1.5 months, respectively; p = 0.03, Figure 3B). When stratified into four groups based on Nectin‐4 and ABC transporter expression, patients with Nectin‐4‐positive and ABC transporter‐negative tumours had the longest PFS (p = 0.002, Figure 3C).

We also analysed the impact of Nectin‐4 and ABC transporter expression in metastatic lesions on PFS after EV therapy in eight patients with available metastatic tissue samples. Patients with Nectin‐4‐positive metastatic lesions had significantly longer PFS than did those with Nectin‐4‐negative lesions (median PFS: not reached vs. 1.7 months, respectively; p = 0.007, Figure 3D). While patients with ABC transporter‐negative metastatic lesions had longer PFS than did those with ABC transporter‐positive lesions (median PFS: not reached vs. 1.9 months, respectively), this difference was not statistically significant (p = 0.10, Figure 3E). When stratified into four groups based on Nectin‐4 and ABC transporter expression, patients with Nectin‐4‐positive and ABC transporter‐negative metastatic lesions had a significantly longer PFS (p = 0.005, Figure 3F).

## 4 DISCUSSION

Our study revealed that, in bladder cancer, progression from non‐muscle‐invasive to muscle‐invasive and metastatic is associated with decreased Nectin‐4 expression and increased ABC transporter expression. Furthermore, higher Nectin‐4 expression and lower ABC transporter expression correlated with longer PFS following EV therapy. With the recent evidence supporting the effectiveness of EV in combination with pembrolizumab as a first‐line treatment for advanced urothelial carcinoma, EV is becoming an increasingly important therapeutic option.^7^ Therefore, the exploration of predictive biomarkers for EV efficacy is an urgent and crucial task for improving patient outcomes. Our findings suggest that both Nectin‐4 and ABC transporters may serve as valuable biomarkers for predicting response to EV therapy, contributing to future optimization of therapeutic strategies.

Although EV is becoming a cornerstone in the treatment of urothelial carcinoma, the mechanisms underlying resistance to ADCs, such as EV, are still not fully understood. Two primary mechanisms have been proposed: i) decreased expression of the target antigen and ii) increased expression of drug efflux transporters.^8^, ^9^ Nectin‐4, the target antigen for EV, is critical for its therapeutic action, and reduced expression of Nectin‐4 has been associated with resistance.^10^ Studies have reported variability in Nectin‐4 expression across different stages and variants of urothelial carcinoma, with a tendency toward reduced expression in muscle‐invasive cancers^11^ and metastatic lesions.^12^ Moreover, the upregulation of ABC transporters, such as MDR1, MRP1 and BCRP, has been implicated in the efflux of cytotoxic payloads,^13^ reducing the effectiveness of ADC therapies, including EV. Our study was focused on these two key mechanisms, highlighting the importance of assessing Nectin‐4 and ABC transporter expression as potential predictors of EV response.

The involvement of ABC transporters, particularly MDR1 (ABCB1), MRP1 (ABCC1) and BCRP (ABCG2), in resistance to traditional chemotherapy has been well established in bladder cancer. MDR1 and MRP1 have been implicated in the development of resistance to cisplatin, a commonly used chemotherapeutic agent for bladder cancer.^5^ These transporters actively efflux chemotherapeutic agents from cancer cells, reducing intracellular drug concentrations and thus limiting their cytotoxic effects. MMAE, the cytotoxic payload of EV, is also recognized as a substrate for ABC transporters,^14^ suggesting that upregulation of these transporters could similarly diminish the efficacy of EV by actively removing the drug from tumour cells. Our findings align with these mechanisms, as we observed an increased expression of ABC transporters in more advanced or metastatic tumours, potentially contributing to EV resistance. The roles of MDR1, MRP1 and BCRP in drug resistance have been well documented, highlighting the importance of these transporters not only in resistance to traditional chemotherapy but also to novel treatments such as ADCs.

The contribution of ABC transporters to ADC resistance extends beyond that in bladder cancer. For example, in preclinical studies of breast cancer cell lines, MDR1 was found to be overexpressed in cells resistant to EV, and inhibition of MDR1 restored drug sensitivity.^15^ Similar mechanisms have been observed in other cancers, such as malignant lymphoma, where cells resistant to brentuximab vedotin, an ADC targeting CD30, exhibit both downregulation of CD30 and increased expression of MDR1.^16^ This suggests that overexpression of ABC transporters is a common mechanism of resistance to ADCs across various cancer types. Our findings contribute to the growing body of evidence that ABC transporters likely play a role in EV resistance in urothelial carcinoma.

Application of several ABC transporter inhibitors, such as tariquidar and elacridar, has been investigated as a potential strategy to overcome multidrug resistance by inhibiting the function of transporters, such as MDR1.^4^, ^13^ While the clinical applications of these inhibitors remain limited, primarily due to toxicity and off‐target effects, preclinical studies have shown that inhibition of ABC transporters can enhance the efficacy of ADCs. For example, the combination of MDR1 inhibitors with ADCs has been shown to reverse drug resistance in cancer cell lines, restoring the cytotoxic activity of the ADCs by preventing drug efflux.^15^, ^17^ These findings highlight the potential of developing combination therapies that include ABC transporter inhibitors to improve the therapeutic outcomes of ADCs, such as EV. Further research is needed to evaluate the safety and efficacy of these combinations in clinical settings.

This study has some limitations that should be acknowledged. First, the sample size was small (20 patients), and the retrospective nature of the study may have introduced biases. Additionally, tissue samples from both primary and metastatic lesions were not available for all patients, limiting the generalizability of our findings. Although it is challenging to obtain tissue from metastatic sites in clinical practice, especially in urothelial carcinoma, this limitation may impact the robustness of the data. Finally, due to the small sample size and limited number of events, we were unable to perform Cox proportional hazards analysis, relying on Kaplan–Meier survival analysis instead. Future studies with larger sample sizes and prospective designs are needed to validate our findings and to explore the utility of Nectin‐4 and ABC transporter expression as predictive biomarkers for EV therapy.

## AUTHOR CONTRIBUTIONS

All authors contributed to the conception and design of the study. The collection of clinical data and survival analysis were performed by Toshiki Kijima. Immunohistochemical staining and pathological analyses were conducted by Atsuko Takada‐Owada. Toshiki Kijima drafted the initial manuscript, and all authors provided feedback on previous versions. All authors have read and approved the final manuscript.

## CONFLICT OF INTEREST STATEMENT

The authors declare that they have no financial or nonfinancial conflicts of interest.

## COMPLIANCE WITH ETHICAL STANDARDS

### ETHICS APPROVAL

This study was approved by the institutional review board of Dokkyo Medical University (approval number: R‐31‐10 J).

## CONSENT TO PARTICIPATE

The informed consent was obtained from all patients before inclusion in the study.

## CONSENT TO PUBLISH

Not applicable.

## Supporting information


**Figure S1.** Frequency of Nectin‐4 and ABC Transporter Expression in Non‐Muscle‐Invasive Cancer, Muscle‐Invasive Cancer and Metastatic Lesions.
Bar graph showing the frequency of Nectin‐4 and ABC transporter (MDR1, MRP1 and BCRP) expression in non‐muscle‐invasive bladder cancer (n = 14), muscle‐invasive bladder cancer (n = 11) and metastatic lesions (n = 8).

## Data Availability

The datasets generated and analysed during the current study are available from the corresponding author upon reasonable request.
