# Supplementary figures and images for: Predictive role of ABC transporters in the efficacy of enfortumab vedotin for urothelial carcinoma
Source: BJUI Compass. 2025 Jan 11;6(1):e488. doi: 10.1002/bco2.488 (PMC11771482; doi:10.1002/bco2.488)

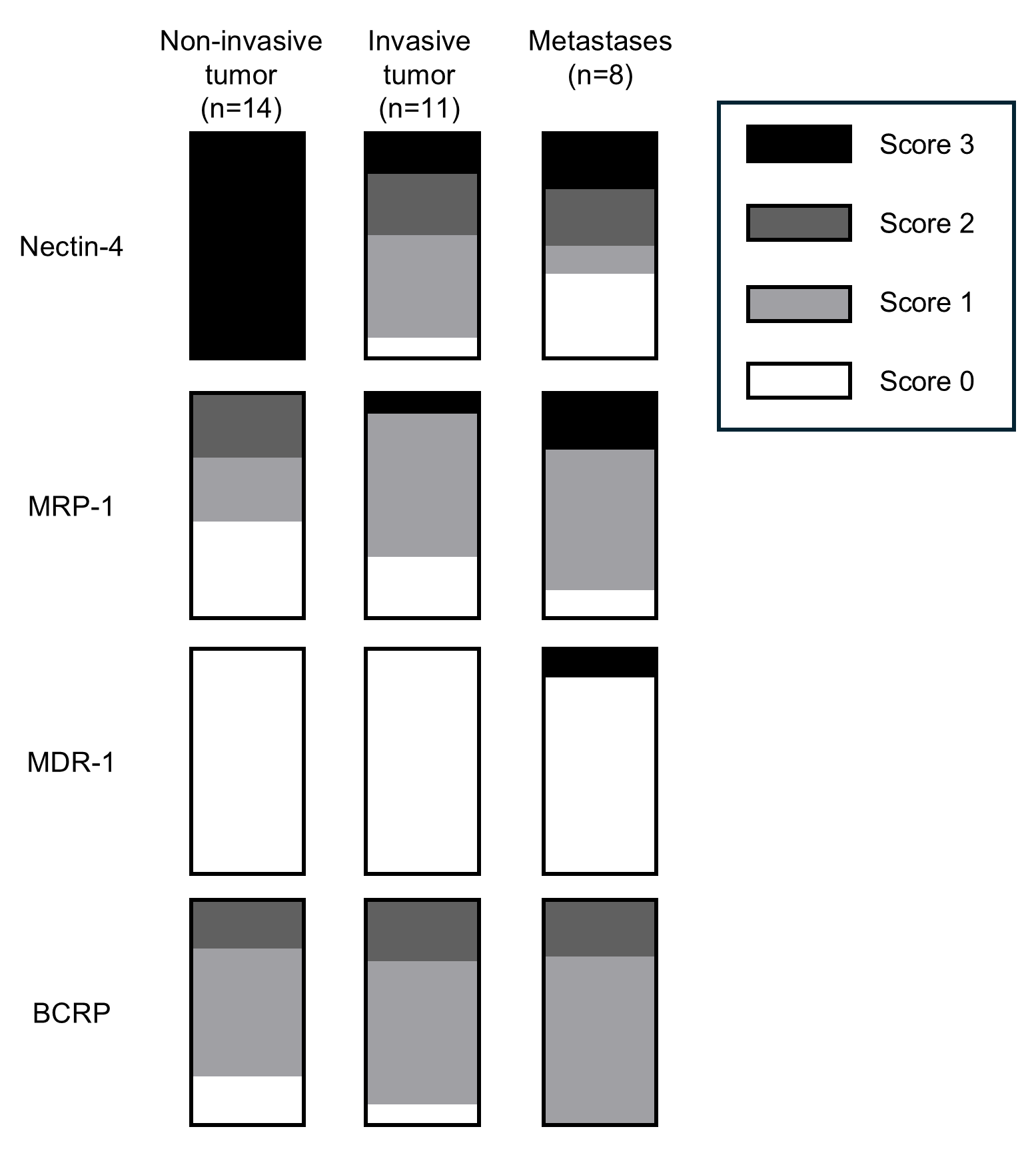

Supplement: Supplementary file 1 — Figure S1. Frequency of Nectin‐4 and ABC Transporter Expression in Non‐Muscle‐Invasive Cancer, Muscle‐Invasive Cancer and Metastatic Lesions. Bar graph showing the frequency of Nectin‐4 and ABC transporter (MDR1, MRP1 and BCRP) expression in non‐muscle‐invasive bladder cancer (n = 14), muscle‐invasive bladder cancer (n = 11) and metastatic lesions (n = 8). [file BCO2-6-e488-s001.tif]
